# Dissection of TALE-dependent gene activation reveals that they induce transcription cooperatively and in both orientations

**DOI:** 10.1371/journal.pone.0173580

**Published:** 2017-03-16

**Authors:** Jana Streubel, Heidi Baum, Jan Grau, Johannes Stuttman, Jens Boch

**Affiliations:** 1 Institute of Plant Genetics, Leibniz Universität Hannover, Hannover, Germany; 2 Department of Plant Genetics, Martin-Luther-Universität Halle-Wittenberg, Halle (Saale), Germany; 3 Institute of Computer Science, Martin-Luther-Universität Halle-Wittenberg, Halle (Saale), Germany; University of Nebraska-Lincoln, UNITED STATES

## Abstract

Plant-pathogenic *Xanthomonas* bacteria inject transcription activator-like effector proteins (TALEs) into host cells to specifically induce transcription of plant genes and enhance susceptibility. Although the DNA-binding mode is well-understood it is still ambiguous how TALEs initiate transcription and whether additional promoter elements are needed to support this. To systematically dissect prerequisites for transcriptional initiation the activity of one TALE was compared on different synthetic *Bs4* promoter fragments. In addition, a large collection of artificial TALEs spanning the *OsSWEET14* promoter was compared. We show that the presence of a TALE alone is not sufficient to initiate transcription suggesting the requirement of additional supporting promoter elements. At the *OsSWEET14* promoter TALEs can initiate transcription from various positions, in a synergistic manner of multiple TALEs binding in parallel to the promoter, and even by binding in reverse orientation. TALEs are known to shift the transcriptional start site, but our data show that this shift depends on the individual position of a TALE within a promoter context. Our results implicate that TALEs function like classical enhancer-binding proteins and initiate transcription in both orientations which has consequences for *in planta* target gene prediction and design of artificial activators.

## Introduction

Plant pathogenic *Xanthomonas* bacteria cause severe losses of crop production worldwide [1]. Their virulence mainly relies on a type-III-secretion system that translocates effector proteins into plant cells [2]. Such proteins interfere with cellular processes and manipulate the plant to the benefit of the pathogen [2]. Transcription activator-like effectors (TALEs) constitute an important group of effectors that manipulate the transcriptome of the host plant [3]. After entering the nucleus TALEs bind to promoter sequences and initiate transcription [3]. TALE-induced plant genes that support pathogen virulence encode transporters for sugar or sulfate, factors that stabilize small RNAs, or transcription factors [4].

**Fig 1:**
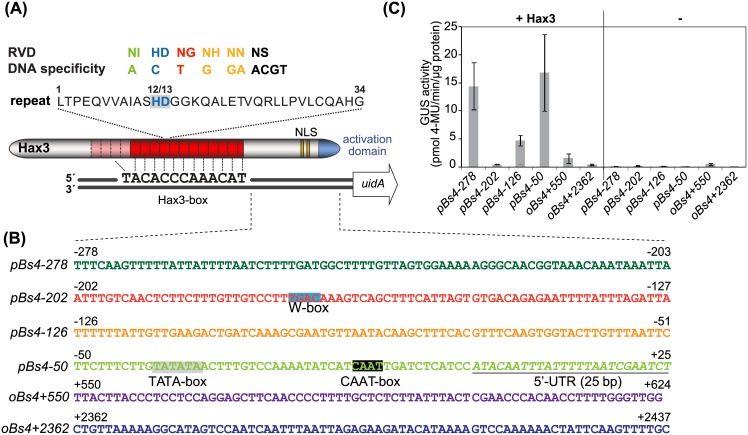
TALE mediated gene activation is dependent on surrounding DNA sequences. (**A**) Model for the DNA-binding mode of TALEs by using the example of Hax3 aligned to the Hax3-box. TALEs contain a central repeat region (red), two nuclear localization signals (NLS) and an acidic activation domain in the C-terminal part. The amino acid sequence of a Hax3 repeat is shown in single letter code. The repeat variable diresidue (RVD) is shaded in grey. Each RVD specifies one nucleotide in the DNA-target sequence. (**B**) Sequence overview of the analysed 75 bp long DNA fragments that originate either from the *Bs4* promoter (*pBs4*, region from -278 bp to +25 bp) or from the *Bs4* open reading frame (*oBs4*). Potential promoter elements that were predicted [37] are labelled in grey (TATA-box) blue (W-box), black (CAAT-box) or italic and underlined (5' untranslated region, UTR). (**C**) TALE-dependent activation of reporter constructs. Each promoter fragment was placed downstream of the Hax3-box and inserted in front of a promoterless *uidA* reporter gene. *Agrobacterium* strains delivering *hax3* under control of the *35S* promoter were co-inoculated into *N*. *benthamiana* leaves along with an *Agrobacterium* strain delivering the respective reporter construct. As negative control, all reporter constructs were inoculated with an empty vector construct (-). The quantitative β-glucuronidase measurement was performed 2 dpi, error bars were calculated on the basis of three independent replicates and represent the standard deviation. (4-MU, 4-methyl-umbelliferone).

DNA association of TALEs is initiated by their N-terminal region whereas the central part of TALEs provides specific binding to matching sequences [3,5,6]. This central DNA binding domain is composed of tandem repetitions of a nearly identical 34 amino acid repeat [3]. Each repeat mainly differs in two-amino acids, the so-called RVD (repeat-variable diresidue), which encodes the specificity for one or several DNA bases (Fig 1a; [7,8]). The TALE repeats form a right handed superhelix that wraps around the DNA double strand [9,10]. Although these repeats can be rearranged to specify practically any given target sequence (called TALE-box or EBE/effector-binding element), a well-balanced presence of so called "strong" and "weak" RVDs is required for efficient activation [11]. A nuclear localization signal (NLS) and an acidic activation domain in the C-terminal part ensure import into the plant nucleus and gene activation, respectively [12–14]. This modular structure and designable DNA-binding specificity has turned TALEs into versatile tools for targeted genome modification and gene regulation in many organisms [15].

Computational analysis of genomic target sites of natural TALEs showed a preferential occurrence in apparent core promoter regions of -300 to +200 bp around the transcriptional start site (TSS; [16]). Previous studies based on the TALEs AvrBs3, AvrXa7, and AvrXa27 showed that they shift the natural TSS of target genes around 40–60 bp downstream of the position at which the TALE is binding the DNA [17–20]. Moving the AvrBs3-box in the *Bs3* promoter to a position further upstream resulted in a concomitant upstream shift of the TSS [19]. These observations led to the impression that TALEs control the onset and the place of transcription functionally analogous to the TATA-binding protein [17]. One host component that is involved in TALE-mediated gene induction has been identified. A rice line that is homozygous for the *xa5* allele exhibits an increased resistance to *Xoo* infection due to a loss of efficiency of TALE-mediated transcriptional initiation [21–23]. *xa5* represents a recessive point mutation of the transcription factor IIA small γ subunit. Apparently, this mutation interferes with the interaction of TALEs with TFIIA and likely accompanying basal transcription-complex components [22–24]. Furthermore, interactions with parts of RNA stabilizing and modifying enzymes have been suggested for TALEs of the PthA family, but so far a functional link is missing [25–28].

In contrast to the already intensively studied DNA-binding mode, the circumstances for successful TALE-mediated transcriptional initiation *in planta* are poorly understood. If the presence of a target sequence alone is sufficient to initiate transcription at any genomic locus or if the target locus has to satisfy additional requirements is still puzzling. Here, we systematically analyze TALE-mediated gene activation potential in different promoter contexts, from different positions or orientations within a promoter, and in a possible cooperative fashion. To dissect whether an effect is dependent on the TALE activation domain or the TALE DNA-binding domain, we replaced the TALE activation domain with a heterologous activation domain and the TALE DNA-binding domain with the Cas9-DNA-binding domain. Our findings suggest that TALEs can not dictate transcriptional activation on their own, but rather rely on further promoter elements. Furthermore, TALEs can support transcription by binding in either forward or reverse orientation, an observation which has not been reported, before. We show that this mode of activation is biologically relevant, because reverse-binding TALEs are able to support virulence of *Xoo* during an infection. This study is significant for understanding TALE-mediated gene activation, and will have implications for target site prediction *in planta*.

## Material and methods

### Bacterial strains and growth conditions

The bacterial strains used in this study were *Escherichia coli* Top10 (New England Biolabs Frankfurt am Main, Germany), *Agrobacterium tumefaciens* GV3101 pMP90 [29] and *Xanthomonas oryzae* pv. *oryzae* (*Xoo*) strains BAI3 [30] and BAI3Δ*talC* [31]. *E*. *coli* cells were cultivated at 37°C in LB, *Xoo* strains at 28°C in PSA and *A*. *tumefaciens* GV3101 at 28°C in YEB medium.

### Plant material and plant inoculations

*Oryza sativa* ssp. *japonica* cv. Nipponbare was grown under glasshouse conditions at 28°C (day) and 25°C (night) at 70% relative humidity (RH). Leaves of 4-week-old plants were infiltrated with a needleless syringe and a bacterial suspension of an optical density (OD) at 600 nm of 0.5 as previously described [32]. Symptoms (watersoaked lesions) were scored 5 days post inoculation (dpi). *Nicotiana benthamiana* plants were grown under 16 h of light, 40–60% RH, at 23°C (day) 19°C (night) in the growth chamber. Leaves of 4- to 6-week-old plants were inoculated with *A*. *tumefaciens* strains using a needleless syringe.

### Construction of artificial TALEs and the dCas9 activator

TALEs were constructed by using the Golden TAL technology as described by Geißler *et al*. (2011). Up to six individual repeats were subcloned in an assembly vector, resulting in a hexa-repeat module. Three of these hexa-repeat-modules were then fused to the N- and C-terminal parts of Hax3 and inserted in an expression vector for *Agrobacterium* or *Xanthomonas*, respectively. This results in a N-terminally fused GFP for the *Agrobacterium* vector and in a C-terminally fused FLAG Epitope in the *Xanthomonas* vector. The dCas9 variant fused to the C-terminal region of Hax3 was used as dCas9 activator and generated as described before [33]. The activity score of the used sgRNAs was analysed by using deskgen (S3 Table).

### β-Glucuronidase (GUS) reporter constructs and GUS activity analysis

β-Glucuronidase assays from plant samples were performed as described by Boch *et al*., 2009. Briefly, the PCR-amplified fragments of the promoters (*Bs4*, *OsSWEET14*) were cloned into pENTR/D-TOPO (Life Technologies GmbH, Darmstadt, Germany) and fused to the *uidA* reporter gene by LR recombination into pGWB3 [34]. To analyze reporter activity, *A*. *tumefaciens* strains delivering TALE constructs and GUS reporter constructs were resuspended in infiltration medium, resulting in an OD_600_ of 0.8, mixed in equal amounts and inoculated into *N*. *benthamiana* leaves. Two dpi, leaf discs were sampled and GUS activities were quantified using 4-methylumbelliferyl-β-D-glucuronide (MUG). Total protein concentrations were determined using Bradford assays. Data were compiled from triplicate samples originating from different plants. Error bars represent the standard deviation.

### RNA isolation and qRT- PCR

Leaves of 4-week-old Nipponbare plants were infiltrated with 10 mM MgCl_2_ or with the different *Xoo* strains using an OD_600_ of 0.5. At 1 dpi, 5 cm segments were harvested and rice total RNA was isolated using the Qiagen RNeasy kit. cDNA was generated from 2 μg RNA using the Fermentas first-strand cDNA synthesis kit (Thermo Fisher Scientific Inc.,Waltham, MA, USA) and real-time PCR was performed using the iCycler (Bio-Rad, München, Germany) as described before (S1 Table) [35]. The amplification efficiency for each primer pair was analyzed using a standard curve plot of a dilution series. cDNA amounts were normalized using actin as a reference gene. The fold change induction was calculated in comparison to leaves treated with the BAI3Δ*talC* mutant by using the ΔΔCt method.

### RNA isolation and 5´RACE

TALE-dependent transcriptional start sites at the *OsSWEET14* promoter were determined by using 5´RACE. Leaf discs were harvested from the same plant material that was used for determining GUS-activity in *N*. *benthamiana* and total RNA was extracted by using the Qiagen RNeasy kit. 1 μg total RNA was used to produce cDNA for 5´RACE by using the SMARTer RACE cDNA amplification kit (Takara Bio, Inc., Shiga, Japan). The 5´ends were amplified by using the universal forward primer and the gene specific primer GUS 5`RACE (S1 Table), cloned, and sequenced.

## Results

### TALE-mediated gene activation depends on flanking DNA sequences

So far it is unknown if TALEs that bind to a certain genomic target site (TALE-box) are sufficient to initiate transcription at that location or if surrounding DNA sequences influence gene induction. To address this, we designed reporter constructs in which the Hax3-box, that is bound by Hax3, a natural TALE from *Xanthomonas campestris* pv. *campestris* [7,36], is placed in front of varying downstream sequences. A 300 bp fragment of the tomato *Bs4* promoter was used. This promoter fragment has a very low basal activity which makes it well suited for transcriptional activation assays [37]. We subdivided this fragment into portions of equal length (75 bp) and placed them downstream of the Hax3-box and upstream of a promoterless *uidA* reporter gene (Fig 1A and 1B). This setup ensured a fixed distance between the TALE-box and the reporter gene open reading frame (ORF; Fig 1A). Additionally, we questioned whether non-promoter regions can trigger TALE-mediated gene expression. Hence, we included two random 75 bp fragments from the *Bs4* ORF in our analysis (Fig 1B). The reporter constructs and the constructs containing *hax3* were co-transformed in *N*. *benthamiana* using *Agrobacterium*-mediated T-DNA transfer. Quantification of the reporter gene activity showed that the three *pBs4* derived fragments *pBs4-278*, *pBs4-126* and *pBs4-50* mediated the strongest Hax3-dependent gene activation (Fig 1C). In contrast, the two *Bs4* ORF sequences *oBs4+550* and *oBs4+2362* and the *pBs4-202* promoter fragment facilitate only a slight or no gene induction by Hax3 (Fig 1C). These findings indicate that TALEs cannot initiate transcription at any genomic location but rely on specific surrounding DNA sequences for efficient gene induction. To get a general idea of other putatively involved DNA-binding factors we predicted potential cis-regulatory motifs by using the program PlantCARE [38] (S1 Fig).

**Fig 2:**
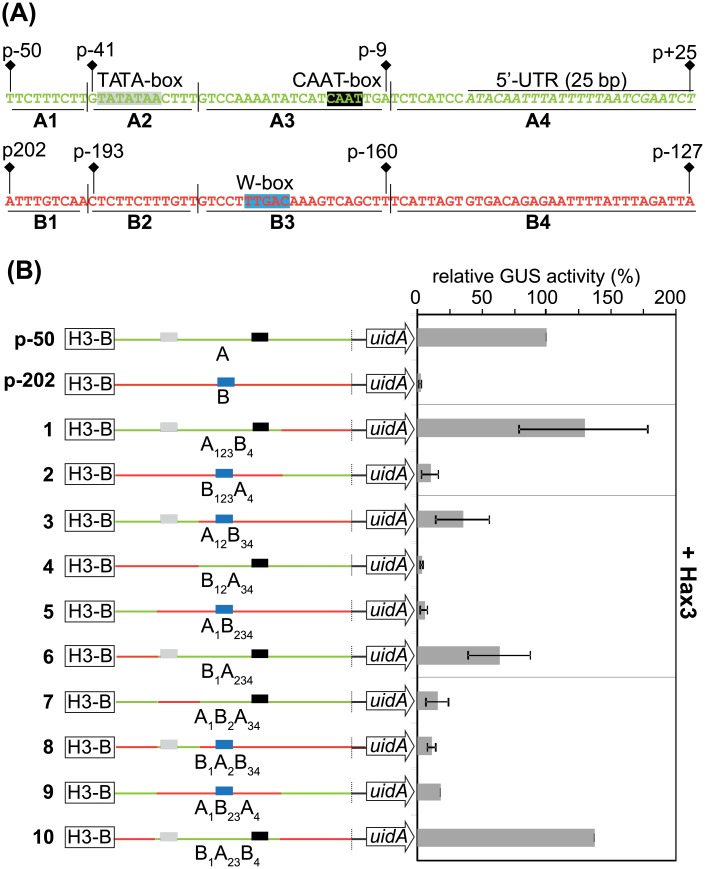
*Bs4* promoter swaps to identify regions that support TALE activity. (**A**) The two 75 bp fragments (A, positive, green and B, negative, red) were subdivided into smaller fragments (A1-4, B1-4). Putative promoter elements that were predicted [37] are labelled in grey (TATA-box), blue (W-box), black (CAAT-box) or italic and overlined (5´UTR). (**B**) Overview of the analysed promoter swaps. The origin of the fused fragments is marked with green and A or red and B, respectively. The fragments were placed downstream of the Hax3-box (H3-B) and upstream of a promoterless *uidA* reporter gene. The dashed line indicates the location of the *attB1* site preceding the *uidA* reporter gene. TALE-dependent activation of the reporter constructs was determined by β-glucuronidase-measurement. *Agrobacterium* strains delivering *hax3* under control of the *35S* promoter were co-inoculated into *N*. *benthamiana* leaves along with an *Agrobacterium* strain delivering the respective reporter construct. The ß-glucuronidase measurement was performed two dpi and calculated as relative activity based on the activity of the reference fragment A (100%). Error bars represent the standard deviation of three independent replicates (4-MU, 4-methyl-umbelliferone).

To further dissect which sequences and potential promoter elements support TALE activity, we chose the fragment *pBs4-50*, that was highly responsive to Hax3, and *pBs4-202*, that was not, and exchanged parts of them (Fig 2A and 2B). We divided the promoter fragments into four parts (A1 to A4 and B1 to B4) and generated promoter-swap constructs fused to the Hax3-box and the *uidA* reporter gene (75bp, Fig 2A and 2B). In comparison to the reference *pBs4-50* only the constructs 1 and 10 showed a comparable or even higher activation by Hax3. This already indicates two findings (i) the reason why *pBs4-50*, but not *pBs4-202* allows TALE-dependent expression is not due to sequences directly downstream of the Hax3-box that might interact with C-terminal parts of Hax3 and (ii) the presence of the 5´UTR in *pBs4-50* is not crucial for robust activity by e.g. influencing mRNA stability. In contrast, all other constructs showed a decreased or no activity (Fig 2B, construct 2–9). Apparently, the predicted CAAT-box, the TATA-box, and the sequence between them are relevant (Fig 2B, construct 6 vs. 10). In summary, these results demonstrate that TALE-boxes which are targeted by TALEs are not always sufficient to initiate transcription. This implies that TALEs, binding to these sequences, do not initiate gene expression on their own. They likely need to bind in proximity to supporting sequences—probably core promoter elements—to ensure efficient gene activation.

### Systematic analysis of TALE-dependent reporter gene activation at the *OsSWEET14* promoter

**Fig 3:**
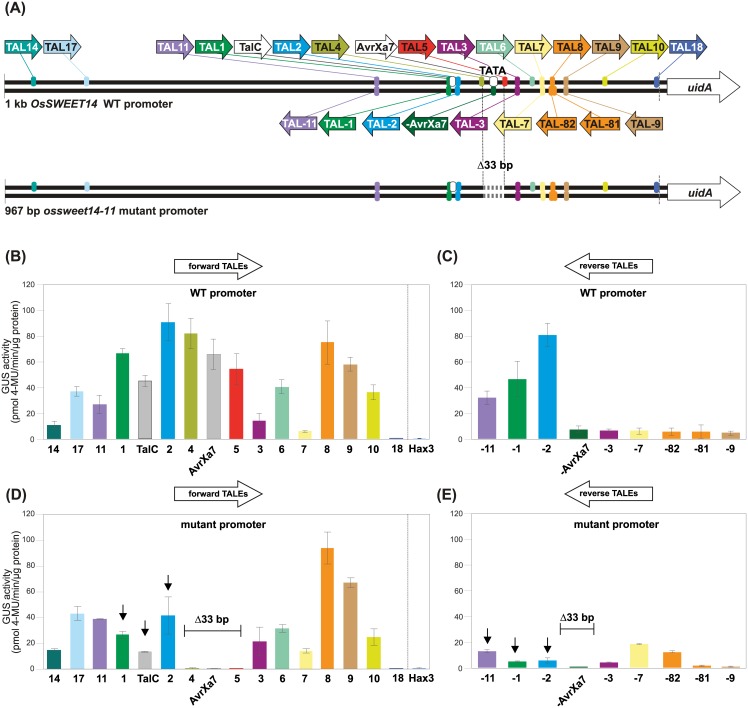
TALEs enhance transcription from diverse positions in the rice *OsSWEET14* promoter in *N*. *benthamiana* in a partially TATA-box dependent manner. (**A**) Overview of the reporter construct containing the *OsSWEET14* wildtype (WT) promoter fragment (1kb upstream of the ATG) and the *OsSWEET14-11* promoter mutant (Δ 33 bp; 967 bp upstream of the ATG; [41]) fused to the promoterless *uidA* reporter gene. The dashed line indicates the location of the *attB1* site preceding the *uidA* reporter gene. The binding sites and binding orientations of artificial TALEs (coloured) and the natural TALEs TalC and AvrXa7 (white) are marked with arrows, the arrowhead indicates the orientation of the activation domain relative to the *uidA* gene. Reverse binding TALEs are labeled with "-" in front of the number. RVD sequences and target sites are listed in S1 Table. **(B)-(E)** Activity of the artificial and natural TALEs. *Agrobacterium* strains delivering TALE constructs under control of the *35S* promoter were co-inoculated into *N*. *benthamiana* leaves along with an *Agrobacterium* strain delivering the reporter construct. The TALE Hax3 that does not bind to the *OsSWEET14* promoter was used as negative control to exclude background promoter activity. The ß-glucuronidase measurement was performed 2 dpi. Error bars represent the standard deviation of three independent replicates (4-MU, 4-methyl-umbelliferone). (**B**) Activity of forward TALEs in combination with the WT reporter. (**C**) Activity of reverse TALEs in combination with the WT reporter. (**D**) Activity of forward TALEs in combination with the mutant reporter. (**E**) Activity of reverse TALEs in combination with the mutant reporter. (D, E) TALEs whose binding sites overlap with the deletion in the mutant promoter are indicated with Δ33 bp. Arrows highlight a strong reduction of TALE activity in comparison to the WT reporter.

To systematically investigate from which promoter positions TALEs can initiate transcription we used a complementary approach in which we kept the promoter sequence constant, but used designer TALEs targeted to different positions. The well characterized rice *OsSWEET14* promoter was used, that is activated by a number of natural and artificial TALEs [20,31,35]. *OsSWEET14* encodes a sugar exporter and represents an important rice virulence target whose TALE-dependent activation supports bacterial virulence [20,31,35,39]. An *OsSWEET14* promoter fragment 1000 bp upstream of the ATG was chosen that comprises possible core and distal promoter elements. The fragment was inserted upstream of a promoterless *uidA* reporter gene which allows a systematic comparison of TALE activities in *N*. *benthamiana*. 14 artificial TALEs with an equal number of 17,5 repeats were built to target various positions within this fragment (Fig 3A; S1 Fig; S2 Table; [40]. By using the TALgetter prediction program [16], all TALEs used in this study have been carefully examined to bind exclusively their desired target sequence and not at other possible locations in the *OsSWEET14* promoter. As control to compare the impact of the varying RVD sequences on TALE activity and to exclude an impact of variable flanking sequences, we first analyzed the corresponding TALE-boxes in the *pBs4-*based reporter construct and verified protein integrity *in planta* (S2 Fig). Next, the TALE constructs were co-delivered together with the *pOsSWEET14* based reporter construct in *N*. *benthamiana* via *Agrobacterium*. The two natural *Xoo* TALEs AvrXa7 and TalC which induce the *OsSWEET14* promoter in the natural *Xoo*-rice interaction [20,31] were used as positive controls and the *OsSWEET14* promoter in combination with a non-matching TALE (Hax3) was used as negative control, respectively. The majority of the analyzed TALEs activated the reporter gene irrespective of their position in the *OsSWEET14* promoter (Fig 3B). This demonstrated that TALEs can drive gene expression from highly different positions in a promoter. TAL7, TAL14, and TAL18 show a particularly low activity (<35% compared to TalC) at the *OsSWEET14* promoter (Fig 3B) although they efficiently induce expression of the *pBs4*- driven reporter gene (S2B Fig). This suggests that their binding sites are not favorable or not accessible in this particular genomic context.

### Artificial TALEs can activate the *OsSWEET14* promoter in reverse orientation

All known TALE targets are based on TALEs binding a promoter in "forward" orientation, with the C-terminal activation domain facing the downstream ORF (Fig 1A). In contrast, classical enhancer elements are often orientation-independent [41–43]. Therefore, we investigated whether TALEs also function in "reverse" orientation *in planta*. Nine TALEs were designed to bind in reverse orientation in the *OsSWEET14* promoter (Fig 3A; S1 Fig; S2 Table) and TALgetter target site predictions verified that these are the only possible binding-sites within the promoter. The two natural *Xoo* TALEs AvrXa7 and TalC were used as positive controls. TALE constructs and the reporter construct were co-delivered in *N*. *benthamiana* via *Agrobacterium*.

Remarkably, the three reversely placed TALEs, TAL-11, TAL-1, and TAL-2, activated the reporter highly efficient with an activity of 71%, 104%, and 179%, respectively, compared to TalC (Fig 3C). We used the highly active TAL-2 to analyze experimentally whether its activation potential was indeed due to its binding in reverse orientation. We designed two reporter constructs based on the *pBs4* reporter system to exclude any *pOsSWEET14*-based effects (S3A Fig). One reporter covers the TAL2 binding site on the forward and the TAL-2 binding site on the reverse strand fused to the minimal *pBs4* promoter and the *uidA* reporter gene. Both TALEs activated this reporter albeit TAL-2 to a significantly lesser extent compared to the *pOsSWEET14* context (S3B Fig). As control, we mutated all five Guanine nucleotides in the upper strand to Adenine (construct TAL2/-2mut). TAL2 should still bind this sequence because its RVD NN recognizes G and A, whereas TAL-2 has HD RVDs matching only to C on the reverse strand (S3 Fig). Accordingly, TAL-2 lost the ability to activate the reporter verifying that TAL-2 in fact binds to the reverse DNA strand (S3B Fig).

The other reverse TALEs resulted in weak activities of 10 to 17% compared to TalC although some of these (i.e., TAL-AvrXa7, TAL-81, TAL-82, TAL-9) target the same region as highly active forward TALEs (Fig 3A, 3B and 3C). All TALEs were active in combination with forward-oriented TALE-boxes upstream of the *pBs4* minimal promoter, and protein levels were comparable, indicating that the RVD composition is not causing the observed variability (S2 Fig).

In summary, the systematic analysis of differentially positioned forward and reverse binding TALEs resulted in three important findings: (i) Some TALEs (TAL11/-11, TAL2/-2 and TAL1/-1) activate transcription in both orientations, suggesting that their DNA target sequences are accessible and allow a flexible interaction with the transcriptional machinery. (ii) The activity of some TALEs (AvrXa7/TAL-AvrXa7, TAL8/-81/-82 and TAL9/-9) is orientation-dependent, suggesting that the reverse orientation in these cases prevented an efficient recruitment of the transcriptional machinery. (iii) Some TALEs do not efficiently activate gene expression irrespective of their orientation, suggesting that either the TALE-box is not accessible within the promoter context or the relative position is not suitable to recruit the transcriptional machinery. The observed activity of reverse binding TALEs breaks the long-standing assumption that TALEs only activate genes in one-direction [16].

### TALEs can synergize to induce transcription at the *OsSWEET14* promoter

**Fig 4:**
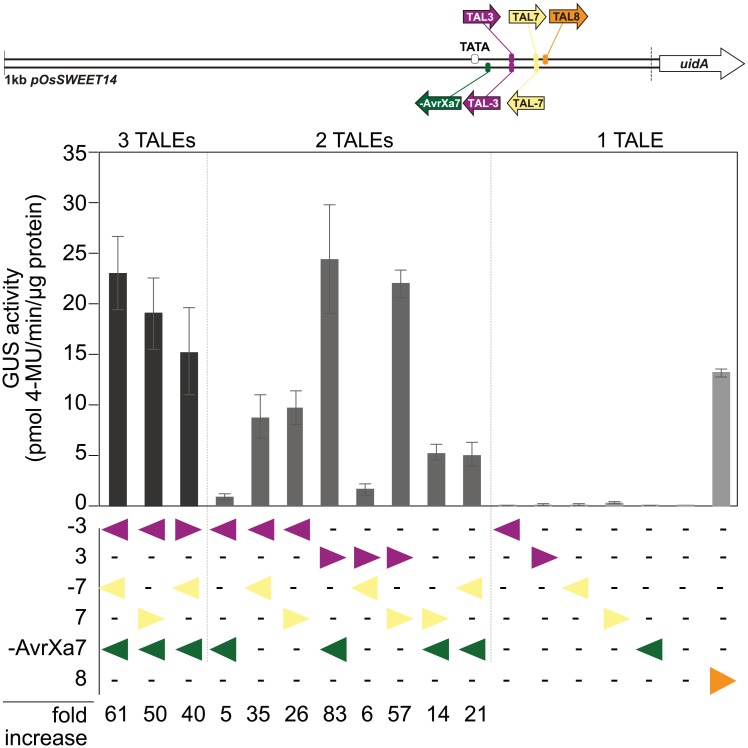
TALEs can synergize for gene induction. *Agrobacterium* strains delivering TALE constructs and the *OsSWEET14*::*uidA* reporter, respectively were mixed at a ratio of 3:1 to a final OD of 1.6. If less than three different TALEs targeting the *OsSWEET14* promoter were used, the remainder was substituted with a *Hax3*-delivering strain not targeting this reporter to keep the total ratio of TALEs constant. The highly active TAL8 was used as positive control. The bacterial mixture was inoculated into *N*. *benthamiana* leaves and GUS measurements were taken 2 dpi, error bars represent the standard deviation of three independent replicates (4-MU, 4-methyl-umbelliferone). Please note that the GUS values of individual low-active TALEs was consistently very low here in comparison to Fig 3, probably due to the higher dilution factor of the reporter construct in this experimental design.

Reverse-binding TALEs seem to be particularly dependent on further promoter elements and we wondered whether they can enhance the capacity of other TALEs to induce transcription. The phenomenon of synergistic gene activation has been described in human cells using TALEs fused to the heterologous VP64 activation domain [44,45], but it is unknown whether the native TALE has a similar potential in plant cells. For this, we used low-activity artificial TALEs from our collection. Individual TALEs or combinations of two or three TALEs and the *OsSWEET14*- reporter were co-transformed into *N*. *benthamiana* and GUS activity was measured. In most cases, combinations of TALEs at the promoter increased transcriptional induction (Fig 4). Even the presence of low activity reverse-binding TALEs can stimulate other TALEs (e.g., TAL3 and -AvrXa7), but this requires that their relative position does not hinder each other e.g., TAL3 and TAL-7 whose activation domains face each other and might spatially block the simultaneous assembly of the transcriptional machinery. Together, this demonstrates that multiple TALEs, including reverse TALEs and low-active TALEs can act synergistically to efficiently induce gene expression in plants.

### The activity of TALEs targeted to reverse oriented sequences is dependent on other promoter elements

Based on our finding that the presence of core promoter regions can influence TALE-mediated gene activation, we aimed to analyze the influence of the TATA-box and surrounding DNA sequences on the activity of TALEs. For this, we used an *OsSWEET14* promoter variant in which 33 bp encompassing the TATA-box were deleted in rice via genome editing [46]. This *ossweet14-11* variant lacks the AvrXa7-box and is thus resistant to *Xoo* strains carrying the TALE AvrXa7 [46]. The edited promoter was amplified from the rice mutant and inserted into the GUS reporter. TALE and reporter constructs were co-transformed into *N*. *benthamiana* and GUS activity was measured.

The deletion of the TATA-box and the surrounding sequences did not significantly influence the activity of the majority of the forward binding TALEs, indicating that an interaction with the transcriptional machinery in these cases is TATA-box independent (Fig 3D). The binding sites of TAL4, TAL5, and Avrxa7 are at least partially deleted resulting in an expected loss of activity (Fig 3d). Surprisingly, the activities of the TALEs that bind directly upstream of the TATA-box, including TAL1, TAL2 and TalC, were reduced by 60, 54, and 70%, respectively, if compared to the activity measured at the wild type promoter (Fig 3B and 3D). This indicates that the activity of TALEs binding to a distinct region directly upstream of the TATA-box is positively supported by this core element and surrounding sequences whereas other TALEs are not. This effect is even more pronounced if the activity of reverse binding TALEs is analyzed. The highly active reverse TALEs TAL-11, TAL-1 and TAL-2 show a strong decrease in their activity of 61, 81 and 91% compared to the WT promoter. This shows that the activity of reverse binding TALEs in particular is highly connected to the presence of supporting DNA-elements like the TATA-box.

### The use of alternative activation and DNA-binding domains, respectively, do not change the relative activation pattern at the *OsSWEET14* promoter

**Fig 5:**
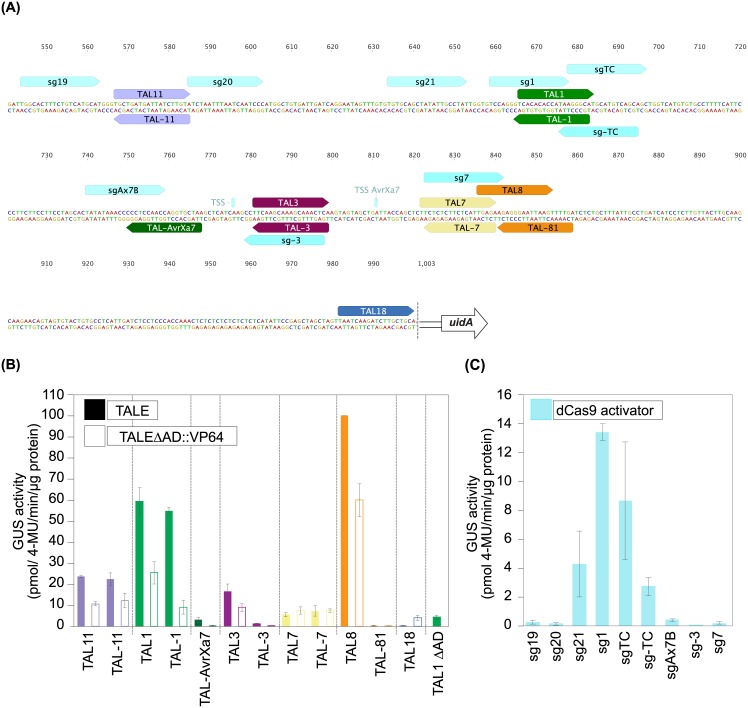
The use of an alternative Activation Domain (AD) or DNA-binding domain (dCas9 activator) does not change the activation pattern of TALEs at the *OsSWEET14* promoter in *N*. *benthamiana*. (**A**) Overview of the binding sites and binding orientation of a selected number of artificial TALEs that were fused to the VP64 AD and the binding sites and binding orientation of the analyzed sgRNAs. The dashed line indicates the location of the *attB1* site preceding the *uidA* reporter gene (**B**) Activity of artificial TALEs (filled bars) in comparison to the TALE-VP64 derivatives (framed bars). *Agrobacterium* strains delivering the TALE constructs under control of the *35S* promoter were co-inoculated into *N*. *benthamiana* leaves along with an *Agrobacterium* strain delivering the reporter construct. TAL1ΔAD was used as an internal control. The ß-glucuronidase measurement was performed 2 dpi. Error bars represent the standard deviation of three independent replicates (4-MU, 4-methyl-umbelliferone). (**C**) The nucleolytically inactive dead Cas9 (dCas9) variant was fused to the C-terminus of Hax3 to generate a dCas9 activator. *Agrobacterium* strains delivering dCas9 activator constructs and the reporter construct that contains the 1 kb promoter fragment of *OsSWEET14* fused to a promoterless *uidA* gene were co-inoculated into *N*. *benthamiana* leaves. GUS measurement was performed 2 dpi, error bars represent the standard deviation of three independent replicates (4-MU, 4-methyl-umbelliferone). Please notice the different scales on the TALE and dCas9 activator graphs indicating that TALEs are more potent activators in this example.

TALE derivatives that were fused to the synthetic VP64 transactivation domain and analyzed in human cell lines showed a highly position and orientation independent activity and activated genes over long distances [44,45,47,48]. TALE-VP16 fusions are in principle known to be functional *in planta* [12], but with a much decreased activity in comparison to natural TALEs [40]. To analyze if the VP64 AD changes the activation pattern relative to the binding position of TALEs *in planta* a subset of our artificial TALEs targeting the *OsSWEET14* promoter was fused to VP64 instead of the TALE AD (Fig 5A). Their gene activation capacity was compared at the *pOsSWEET14*::*uidA* reporter construct following transformation into *N*. *benthamiana*. The overall activity of the VP64-fused TALEs was lower than the activity of the TALEs containing their native AD, likely because VP64 is not completely compatible with the plant transcriptional machinery. Importantly, the use of VP64 instead of the TALE AD did not profoundly change the relative activation level of the analyzed TALEs at their respective positions (Fig 5B). This indicates that the TALE AD functions in a manner that is analogous to the VP64 AD in plant cells.

Although TALEs employ a highly flexible DNA binding domain their access to DNA can be inhibited by methylated cytosines or nucleosomes occupying the sequences [49,50]. The *Streptococcus pyogenes* Cas9 is a non-related DNA-binding protein that is guided to target sequences by a mechanism that is unrelated to TALEs [51]. DNA-binding of SpCas9 is directed by a guide RNA that forms base pairing to 20 nucleotides in the target sequence and is therefore not affected by methylation [51]. To test whether the TALE DNA-binding domain or its activation domain is responsible for the orientation-independent gene activation we analyzed the TALE-activation domain in the context of the Cas9-DNA binding platform. We fused the catalytically inactive "dead Cas9" (dCas9, D10A; N863A; [52] lacking nuclease activity to the C-terminus of Hax3 to generate a dCas9 activator that is functional in plants [33,53]. To compare the activity of TALEs and the dCas9 activators we designed sgRNAs (single guide RNAs) that bind in proximity to existing TALE-boxes (Fig 5C; S3 Table). T-DNAs producing TALE and Cas9-activator, respectively, were co-transformed with the reporter construct containing the *OsSWEET14* promoter into *N*. *benthamiana* and reporter gene activation was determined. Those sgRNAs that hybridized to the region neighboring the highly active TAL1 and TalC mediated strongest reporter gene activation (Fig 5C). Taken together, the use of an alternative DNA binding domain identified the same promoter region upstream of the TATA-box as highly suitable for activation by the TALE-AD.

### TALEs control the transcriptional start site dependent on their position

**Fig 6:**
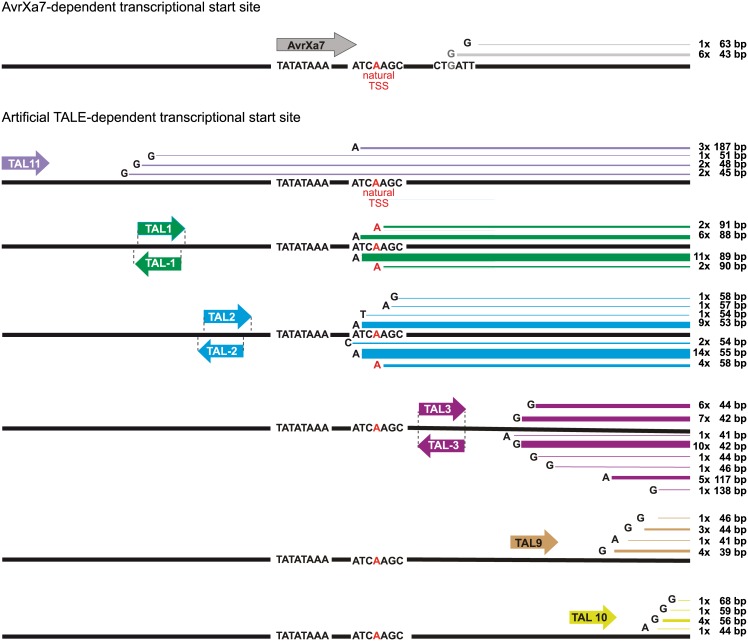
Artificial TALEs do not always shift the natural Transcriptional Start Site (TSS) at the *OsSWEET14* promoter in *N*. *benthamiana*. Overview of analyzed artificial and natural TALEs that bind to different positions in the *OsSWEET14* promoter. The natural *OsSWEET14* TSS in rice is marked with a red "A", and the AvrXa7-induced TSS is marked with a grey "G" [20]. *Agrobacterium* strains delivering the *35S*-controlled TALE constructs were co-inoculated into *N*. *benthamiana* leaves along with an *Agrobacterium* strain delivering the reporter construct. 1 dpi leaf discs were harvested and total RNA was extracted. cDNA was produced and used for 5' RACE. The first nucleotide of each identified TSS is labeled with a capital letter. The distance of the TSS to the 3´end of forward-orientated TALE target sequences and to the 5´end of reverse-orientated TALE target sequences as well as the number of analyzed clones ("x") is indicated to the right. TSSs that overlap with the natural *OsSWEET14* TSS in rice are marked in red.

Previous studies indicated that TALEs can shift the transcriptional start site (TSS) of a plant gene [17–20]. Since some of them bound to sequences overlapping the TATA-box, it is not clear whether the TSS shift in these previous examples is based on the action of the TALE or by spatially blocking the TATA-box. Hence, we aimed to systematically analyze TALE dependent TSS shifts in the *OsSWEET14* promoter by using our collection of differentially positioned artificial TALEs. The TALE constructs were co-transformed together with the reporter construct into *N*. *benthamiana* followed by RNA extraction and 5´RACE. The AvrXa7-dependent TSS at the *OsSWEET14* reporter construct in *N*. *benthamiana* resembled the one following *Xanthomonas*-mediated delivery in rice indicating that both systems are comparable (Fig 6; [20]).

The TALEs that were located downstream of the TATA-box (TAL3/-3, TAL-9, TAL-10; Fig 6) shifted the *OsSWEET14* TSS to positions around 40–60 bp downstream of the respective TALE-box which is in concordance to previous observations (Fig 6; [17–20]. Interestingly, the TSS for TAL3 and TAL-3 are comparable although the activation domain which is supposed to be the platform to assemble the initiation complex is located in a flipped orientation. TALEs binding upstream of the TATA-box (TAL2/-2, TAL1/-1 and TAL11) on the other hand show a different response. TAL2 and TAL-2 initiate transcription around 50–60 bp downstream of their target site which mainly resembles the natural *OsSWEET14* TSS (Fig 6). Although the binding site of TAL1 and TAL-1 is located further upstream it primarily triggers the same TSS as TAL2 and TAL-2 (Fig 6). This suggests that the TSS in presence of TALEs that bind in a certain region may not primarily be dictated by the position or orientation of the TALE itself but by the proximity to other active promoter-elements e.g. the TATA-box. In contrast, TAL11, which binds even further upstream of the TATA-box, again resulted in a TSS 40-60bp downstream of its binding site and additionally in the natural TSS (Fig 6). These results show that TALEs can trigger transcription in two ways. Either they directly influence the TSS, suggesting a direct role in the assembly of the pre-initiation complex (PIC) or they support other promoter elements e.g. the TATA-box which recruit the PIC.

### Artificial reverse binding TALEs restore the virulence of the BAI3Δ*talC* mutant in rice

To analyze whether reverse binding TALEs can activate expression of target genes in a natural infection, a subset of artificial TALEs was introduced into the *Xoo* mutant BAI3Δ*talC* ([31]. This mutant is deficient in the major virulence factor *talc* which targets *OsSWEET14*. Consequently, this strain fails to induce *OsSWEET14* and does not cause disease symptoms [31]. Complementation of this *Xoo* mutant strain with *talC* or artificial TALEs that target *OsSWEET14* restored *OsSWEET14* induction and virulence [31,35]

**Fig 7:**
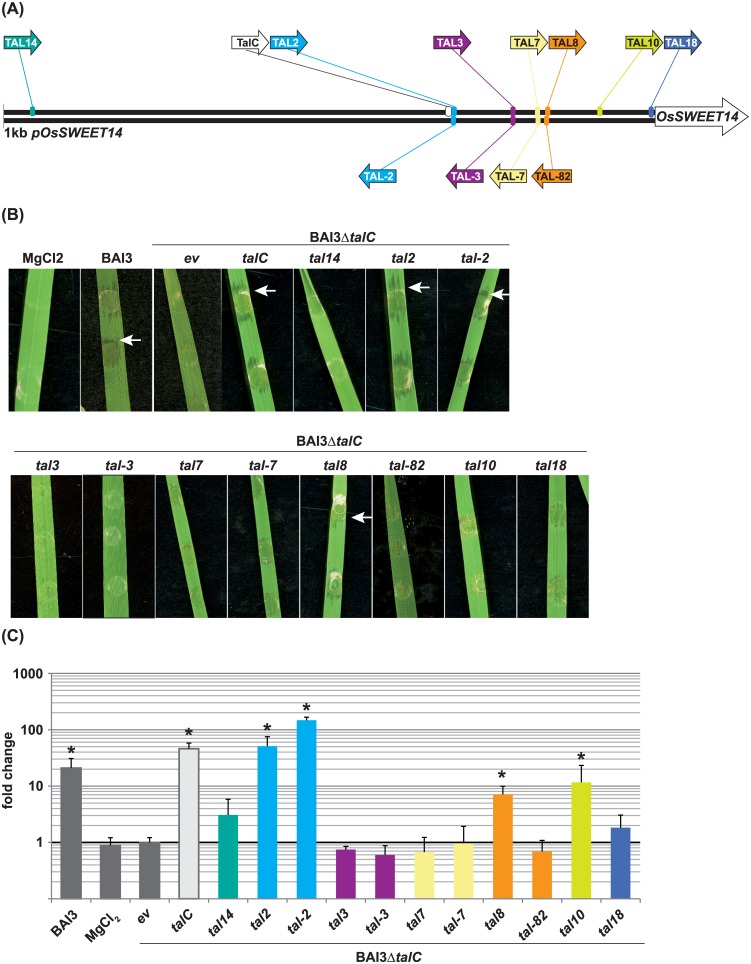
Reverse binding artificial TALEs can activate *OsSWEET14* expression in a natural *Xoo*-rice infection. (**A**) Overview of the binding sites and binding orientation of analyzed artificial TALEs (coloured arrows) and of the natural TALE TalC (white arrow). (**B**) Reverse- and forward-oriented TALEs can both support disease development of *Xoo* in rice. Leaves of *Oryza sativa* cv. Nipponbare were inoculated with the *Xoo* strains BAI3, BAI3Δ*talC* or BAI3Δ*talC* carrying an empty vector plasmid (*ev*), *talC* or an artificial *TALE* on a plasmid. Inoculation of rice leaves with MgCl_2_ served as negative control. Pictures of phenotypes were taken 5 dpi. Water soaking symptoms are marked with a white arrow. (**C**) *OsSWEET14* expression levels following *Xoo* infection. Leaves of *Oryza sativa* cv. Nipponbare were inoculated with the same *Xoo* strains as in (**B**). 1 dpi leaf material was harvested and the transcript levels of *OsSWEET14* were determined by quantitative reverse transcription polymerase chain reaction (qRT-PCR). The error bars indicate the standard deviation of three biological replicates. The fold-change of *OsSWEET14* expression was calculated based on the negative control BAI3Δ*talC* + *ev*. An asterisk (*) indicates a significant increase in *OsSWEET14* expression calculated with the students *t*-Test.

*Xoo* BAI3Δ*talC* was complemented with artificial TALEs from our collection, and *talC*, respectively (Fig 7A). The strains were inoculated into the rice variety Nipponbare, and virulence symptoms were documented in comparison to the mutant strain without any *OsSWEET14*-targeting TALE (Fig 7B). To test TALE-dependent activation of *OsSWEET14*, qRT-PCR was performed in parallel. The forward-binding TALEs TAL2, TAL8, and TAL10 efficiently induced *OsSWEET14* and supported the development of disease symptoms. Moreover, also the highly active reverse binding TALE TAL-2 resulted in a gain of virulence and significant increase in *OsSWEET14* expression (Fig 7B and 7C). However none of the forward or the reverse binding TALEs TAL14, TAL3/-3, TAL7/-7, and TAL-82, which had a low activity in the *N*. *benthamiana* transient system, restored the virulence of BAI3Δ*talC* or activated the expression of *OsSWEET14* significantly (Fig 7B and 7C). One of the TALEs, TAL10, did induce *OsSWEET14* expression, but did not lead to a gain of virulence. Instead, it led to the formation of dark brown lesions around the inoculation spot, suggesting that this TALE triggers a collateral defence reaction in parallel which blocks the development of virulence symptoms (Fig 7B and 7C). In summary, TALEs can activate a target gene from different promoter positions and via binding in the reverse orientation also in the biologically relevant natural infection system.

## Discussion

TALEs are versatile virulence factors that have been evolved by the pathogen to target different plant promoter sequences and induce a variety of target genes. The unique modular TALE DNA-binding domain has been well studied, but how the host transcription machinery is recruited and whether further promoter elements are needed is still largely unknown. Protein interaction screens to identify host components have yielded only few candidates, and those shed little light on the gene induction requirements [23,25,27,54–56]. Instead, we here used a set of functional studies to gain first insights into the conditions under which TALEs induce gene expression.

Our reporter gene induction experiments demonstrate that the presence of a TALE binding site alone can not trigger transcription, instead, a specific DNA context is required. This indicates that TALEs function analogous to enhancer-binding proteins that require the basal transcription machinery for their action and function as control agents for gene expression. Previous studies have revealed a binding preference for natural TALEs at -300 to +200 bp around the TSS of a target gene, a region that typically includes core promoter elements [16,57,58]. One of such elements apparently supporting TALE-mediated gene activation in some of our experiments, is the TATA-box. Interestingly, several natural TALE-boxes overlap with the TATA-box of their respective target genes [16,20,31,59–64]. This finding led to the assumption that TALEs functionally replace the TBP [17]. According to our present data, this is presumably not the case. Instead TALEs can benefit from the presence of a TATA-box. In *Oryza sativa* ssp. *japonica* only around 19% of the promoters contain a TATA-box [65,66]. Indeed, some TALE-activated promoters (*Xa23* [67,68], *OsSWEET2b*, *OsSWEET4* [35], *pAGT582-1* [69], and the fragments *pBs4-278*, *pBs4-126* and *oBs4+550* used in this study) lack a prototype TATA-box [65,70]. This shows that the presence of a TATA-box is not absolutely required for TALE-dependent induction, but can be substituted for by certain other sequences.

Using the prominent TALE virulence target *OsSWEET14* [20,31] from rice, we could further show that TALEs function from many different positions within a given promoter, but not equally well. Although individual artificial TALEs have been used before to induce promoters a quantitative comparison of multiple TALE positions within a native promoter context has not been done, so far. The variable efficiency of TALEs could be caused by several reasons: sequences are blocked by other proteins or nucleosomes [50], TALEs do not bind equally well because of their RVD composition [11], methylation of target sequences interferes with RVD-base recognition [49,71–73], or by the relative position of TALEs to other transcription factors. We surveyed available data to identify occupied and accessible DNA regions in the *OsSWEET14* promoter (S4 Fig). Although these data do not give a consistent picture, the region upstream of the TATA-box should in principle be accessible for TALEs. To exclude factors like RVD composition, DNA methylation, and other effects specific for the TALE-DNA interaction, we employed designer activators as fusions between dCas9 and the C-terminal domain of TALEs [33]. Comparison of a collection of sgRNAs to target the dCas9-activator to different positions within the *OsSWEET14* promoter revealed that they are also position-dependent and that it is not the DNA-binding domain, but rather the position of the TALE activation domain within the promoter context that dictates the efficiency of the activator. Although classical enhancers are typically regarded as being highly independent in their positioning relative to a given promoter, this is not unambiguously the case [41,42]. In fact, re-positioning of such enhancer elements within promoters demonstrated that they also show a different activity at different positions [74–76]. We postulate that TALEs can in principle function from different positions within a promoter, thus functionally resemble classical enhancer-binding proteins.

It has been reported earlier that TALEs shift the transcription start site (TSS) to a position 40–60 bp downstream of their binding position upon gene activation [17–20], but do not do so in a synthetic promoter with multiple inserted TALE-boxes (and TATA-boxes) [77]. The analysis of our collection of TALEs targeting the *OsSWEET14* promoter now suggests, that the TALE-dependent TSS shift does not depend on the TALE alone, but depends on the presence of additional promoter elements. TALEs positioned in a region closely upstream of the natural TATA-box supported the natural TSS, whereas TALEs positioned further upstream or downstream triggered novel TSS. We envision that TALEs are enhancer-binding proteins that define their own binding position according to their modular DNA-binding domain, but at the same time, they directly cooperate with and recruit the transcription machinery.

In a breakthrough study one of these interactions could recently be shown [23]. TALEs interact with the gamma subunit of the basal transcription factor TFIIA [23], which is well known to interact with activation domains of several activators similar to the one from TALEs, belonging to the family of acidic activation domains (e.g. VP16, Zta and Gal4 [78–82]). Indeed, when we replaced the native acidic activation domain of TALEs with the VP64 activation domain, these activators contained a similar relative activity profile, with their highest activity at comparable positions to native TALEs at the *OsSWEET14* promoter. This shows again that the position within the promoter dictates the overall efficiency of the activator and that the activation domain can functionally be replaced by related ones. Our TALE-VP64 fusions still contained most of the C-terminal domain of native TALEs and the portion that interacts with TFIIAy [23]. This domain might therefore have facilitated the interaction to the basal transcription machinery, and caused the comparable activity profiles along the different promoter positions. Because the region upstream of the TATA-box in the *OsSWEET14* promoter is so well suited for activators, we propose that our TALE-positioning screen actually identified a region spanning a potential natural enhancer element that regulates the expression of *OsSWEET14* in rice [83,84].

We were highly surprised to realize that TALEs also function when bound in a reverse orientation relative to the open reading frame. This has, so far, not been considered for the identification of TALE targets and the reverse binding mode will be an important implementation to identify novel TALE virulence targets. It further points out that the orientation, relative to the TSS, of the TALE activation domain, which is interacting with host components, does not play a role under certain, but not all, conditions. This observation further suggests that TALEs may initiate transcription bidirectionally. A bidirectional transcription describes a situation in which—originating from one promoter—two divergent transcripts are initiated [85,86]. This phenomenon has frequently been observed for many promoters of human and animal origin and increasingly also for plants [76,86–93]. During the review process of our study another manuscript was published which supported our findings that TALEs function when bound in reverse orientation and initiate transcription bidirectionally [94]. In this work, the authors suggest that forward and reverse binding TALEs function equally well [94]. In contrast, in our experimental setup most reversely placed TALEs did not induce transcription to the same level as forward placed ones (Fig 3 an S3 Fig). This suggests that although the bidirectional transcription is a general feature of TALEs it is possibly restricted by surrounding promoter sequences. Therefore, we propose that TALEs only function in reverse orientation if placed in defined promoter regions that allow bidirectional transcription, e.g., the region upstream of the TATA-box in the *OsSWEET14* promoter. This indicates that both orientations are not equally well suited to induce gene expression and that TALEs prefer the forward orientation. Possibly, in reverse orientation, the TALE protein itself is blocking the path of the polymerase and an efficient establishment of a novel TSS can only occur downstream of the TALE. This is supported by our observation that both, forward- and reverse-binding TALEs upstream of the TATA-box trigger the same TSS downstream. This further suggests that TALEs bound in either one of the orientations can activate already present, but paused polymerase II complexes [95].

Comparing the activity of our TALE collection at the wild-type and a promoter-derivative lacking the TATA-box and surrounding sequences revealed that those TALEs that are located closely upstream of the TATA-box function less well, whereas further upstream positioned TALEs and TALEs downstream of the TATA-box are not influenced. This indicates that TALEs can either use existing elements or use novel downstream promoter sequences if they are suitable. In the latter case, the TSS is changed. This observation is consistent with our promoter-swap experiments which revealed that the region downstream of the TALE is crucial for TALE-dependent gene activation. Intriguingly, the activity of the reverse-oriented TALEs in particular is negatively impacted by the removal of the TATA-box. This indicates that transcription initiated by reverse TALEs is especially dependent on the presence of potent other promoter elements. This restriction is likely the reason why so far most natural TALEs bind in forward orientation at their respective target promoters.

Our combination of several weakly active TALEs targeted to either forward or reverse binding sites in the *OsSWEET14* promoter revealed that TALEs can act synergistically in plants. This feature was only shown in human cells before, where TALE derivatives with a truncated C-terminus were fused to the VP64 activation domain [44,45,96]. Interestingly, the synergistic effect was only observed if TALEs do not spatially hinder each other. Our results further suggest that TALEs *in planta* may not only act synergistically with each other but are further supported by promoter elements which are possibly occupied by host transcription factors (e.g. the TATA-box). Whether the synergistic activity of TALEs is based on increased chromatin remodeling or other effects remains to be elucidated.

Our systematic analysis of variably positioned TALEs shows that they act in principle as enhancer-binding proteins. When placing designer TALE activators in promoters to trigger efficient target gene activation one should consider the presence of existing promoter elements, e.g. the TATA-box, and the expected possible shift of the TSS. The novel reverse binding mode of TALEs now allows for the detection of formerly overlooked virulence targets in host plants.

Therefore, we have added the option to search for reverse-binding TALEs in the TALE-target prediction programs TALgetter [16], and the feature is also implemented in the alternative programs TALVEZ and TALE-NT 2.0, respectively [97,98]. Forward and reverse binding TALEs will furthermore be excellent tools to tackle fundamental questions of gene induction in plants.

## Supporting information

S1 Fig PlantCARE prediction of cis-regulatory motifs in the different *Bs4*-derived fragments. Sequence overview of the analysed 75 bp long DNA fragments that originate either from the *Bs4* promoter (*pBs4*, region from -278 bp to +25 bp) or from the *Bs4* open reading frame (*oBs4*). Potential cis-regulatory motifs according to the PlantCARE (Lescot *et al*., 2002) prediction were marked with different colors on either the forward or the reverse strand of the fragments. The potential function of those motifs according to PlantCARE was noted behind the name of the motif.
(PDF)

S2 Fig *OsSWEET14* promoter sequence and TALE occupancy. Overview of the *OsSWEET14* promoter fragment 1kb upstream of the ATG. The binding sites and binding orientation of artificial TALEs (coloured) and the natural TALEs TalC and AvrXa7 (white) is marked with arrows. Reverse binding TALEs are labeled with "-" in front of the number. The 33 bp deletion in the sweet14-11 promoter is indicated.
(PDF)

S3 Fig Comparison of artificial TALEs using *pBs4* reporter constructs and western-blot. **(A)** Schematic overview of the reporter constructs. To compare the activation potential of each TALE independent of their position or orientation in the *OsSWEET14* promoter their 18 bp TALE-boxes were inserted in forward orientation in front of the 75 bp *pBs4* minimal promoter and a promoterless *uidA* reporter gene. **(B)** Activity of artificial and natural TALEs. *Agrobacterium* strains delivering the *35S*-controlled TALE constructs and reporter constructs, respectively, were co-inoculated into *N*. *benthamiana* leaves. The TALE Hax3 and its Hax3-box reporter were used as positive control. The quantitative β-glucuronidase measurement was performed 2 dpi. Error bars were calculated on the basis of three independent replicates (4-MU, 4-methyl-umbelliferone). **(C)** Detection of TALE proteins in *N*. *benthamiana*. In parallel to the β-glucuronidase assay, six leaf discs of the inoculated area were harvested to analyze protein levels of the TALEs in *N*. *benthamiana*. SDS-PAGE followed by immunoblotting with an anti-GFP antibody directed against the N-terminal GFP that was fused to all analyzed TALEs shows a stable protein production. The expected size of artificial TALEs with 17.5 repeats and Hax3 with 11.5 repeats is indicated in kDa. The expression of GFP served as positive control, the sample with not-inoculated plant material (empty) as negative control.
(PDF)

S4 Fig Specificity of the reverse binding TAL-2. **(A)** Overlapping target sequences of the TALEs TAL2 and TAL-2 within reporter constructs. The TAL2/-2-box encompassing both TALE-boxes and the TAL2/-2mut-box with point mutations (red letters) that interfere only with binding of TAL-2, but not TAL2 were fused to the minimal promoter (75 bp *Bs4*) and a promoterless uidA reporter gene. **(B)** The activities of the TALEs were determined by quantitative β-Glucuronidase (GUS) measurement in *N*. *benthamiana*. *Agrobacterium* strains delivering the *35S* controlled TALE constructs and the corresponding reporter constructs, respectively, were co-inoculated into *N*. *benthamiana* leaves. The TALE Hax3 and its Hax3-box reporter construct were used as control. GUS measurement were performed 2 dpi, error bars were calculated on the basis of three independent replicates (4-MU, 4-methyl-umbelliferone).
(PDF)

S5 Fig Epigenetic landscape of the *OsSWEET14* promoter. The 1 kb fragment of the *OsSWEET14* promoter including the location of TALE-boxes (coloured boxes) was aligned to DNase I, MNase or bisulfite sequencing profiles indicating occupied promoter regions. DNase I and MNase profiles for rice leaves were downloaded from PlantDHS (http://plantdhs.org/Download, Zhang et al., 2015) in bigwig format. In addition, DNase I hypersensitive sites (DHS) and nucleosome tracks were downloaded from PlantDHS in gff format. DNase I reads of rice seedlings from (Zhang et al., 2012) where downloaded from NCBI Sequence Read Archive (SRA), accession SRX038423, and mapped to the rice MSU7/TIGR7 genome using bowtie2 (Langmead & Salzberg, 2012) with seed length 15 and at most 1 seed mismatch. MNase-Seq data from (Zhang et al., 2015b) of length 75 bp (SRR1536134) and 36 bp (SRR1536112) where also downloaded from SRA and mapped using bowtie2 with identical parameters. Bisulfite sequencing (BS-Seq) data of rice panicles from (Li et al., 2012) were downloaded from SRA (SRR037418, SRR037419, SRR037421, and SRR037422) and nucleotide-wise methylation percentages where determined using Bismarck (Krueger & Andrews, 2011) with bedGraph output.
(PDF)

S1 Table Oligonucleotides used in this study. (DOCX)

S2 Table Artificial TALEs, their RVD sequences, and target DNA sequences. (DOCX)

S3 Table sgRNA sequences and deskgen score. (DOCX)

S1 References (DOCX)
