# Correction: Dissection of TALE-dependent gene activation reveals that they induce transcription cooperatively and in both orientations

**DOI:** 10.1371/journal.pone.0175653

**Published:** 2017-04-06

**Authors:** Jana Streubel, Heidi Baum, Jan Grau, Johannes Stuttmann, Jens Boch

The fourth author’s name is spelled incorrectly. The correct name is: Johannes Stuttmann. The correct citation is: Streubel J, Baum H, Grau J, Stuttmann J, Boch J (2017) Dissection of TALE-dependent gene activation reveals that they induce transcription cooperatively and in both orientations. PLoS ONE 12(3): e0173580. doi:10.1371/journal.pone.0173580

## References

[1] Streubel J, Baum H, Grau J, Stuttman J, Boch J (2017) Dissection of TALE-dependent gene activation reveals that they induce transcription cooperatively and in both orientations. PLoS ONE 12(3): e0173580 doi:10.1371/journal.pone.0173580 28301511 10.1371/journal.pone.0173580 PMC5354296

