# Supplementary figures and images for: Dissection of TALE-dependent gene activation reveals that they induce transcription cooperatively and in both orientations
Source: PLoS One. 2017 Mar 16;12(3):e0173580. doi: 10.1371/journal.pone.0173580 (PMC5354296; doi:10.1371/journal.pone.0173580)

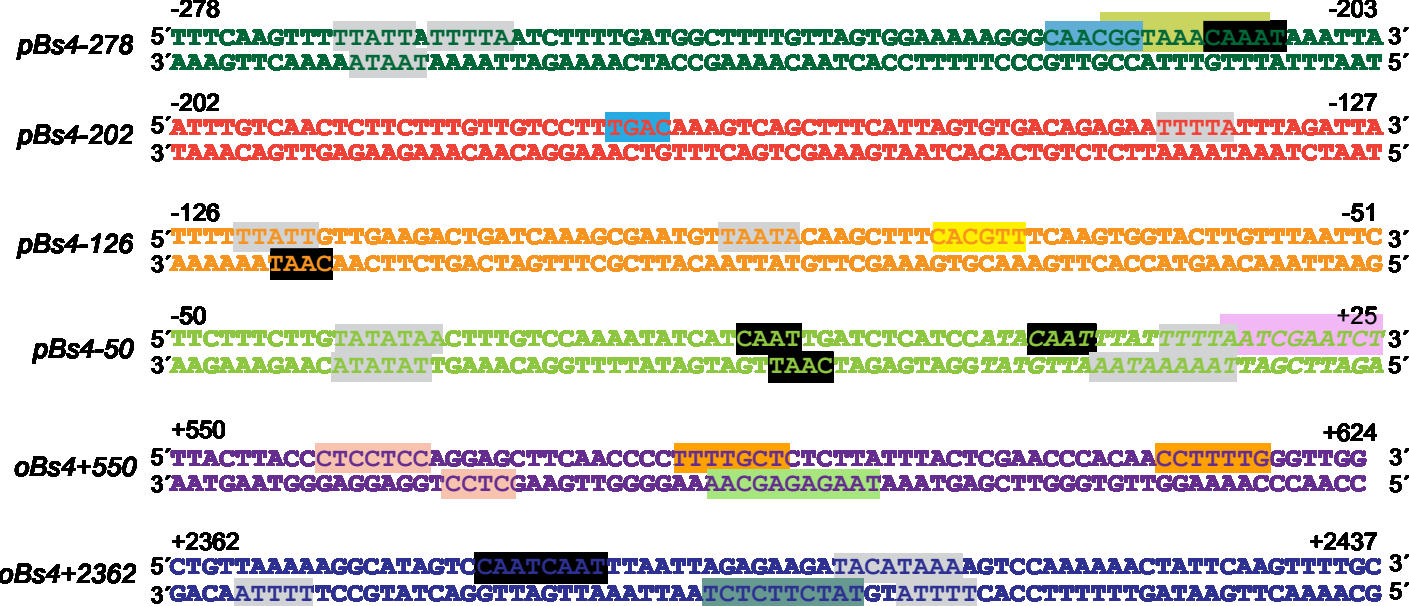

Supplement: S1 Fig — Sequence overview of the analysed 75 bp long DNA fragments that originate either from the Bs4 promoter (pBs4, region from -278 bp to +25 bp) or from the Bs4 open reading frame (oBs4). Potential cis-regulatory motifs according to the PlantCARE (Lescot et al., 2002) prediction were marked with different colors on either the forward or the reverse strand of the fragments. The potential function of those motifs according to PlantCARE was noted behind the name of the motif. (PDF) [file pone.0173580.s001.pdf]

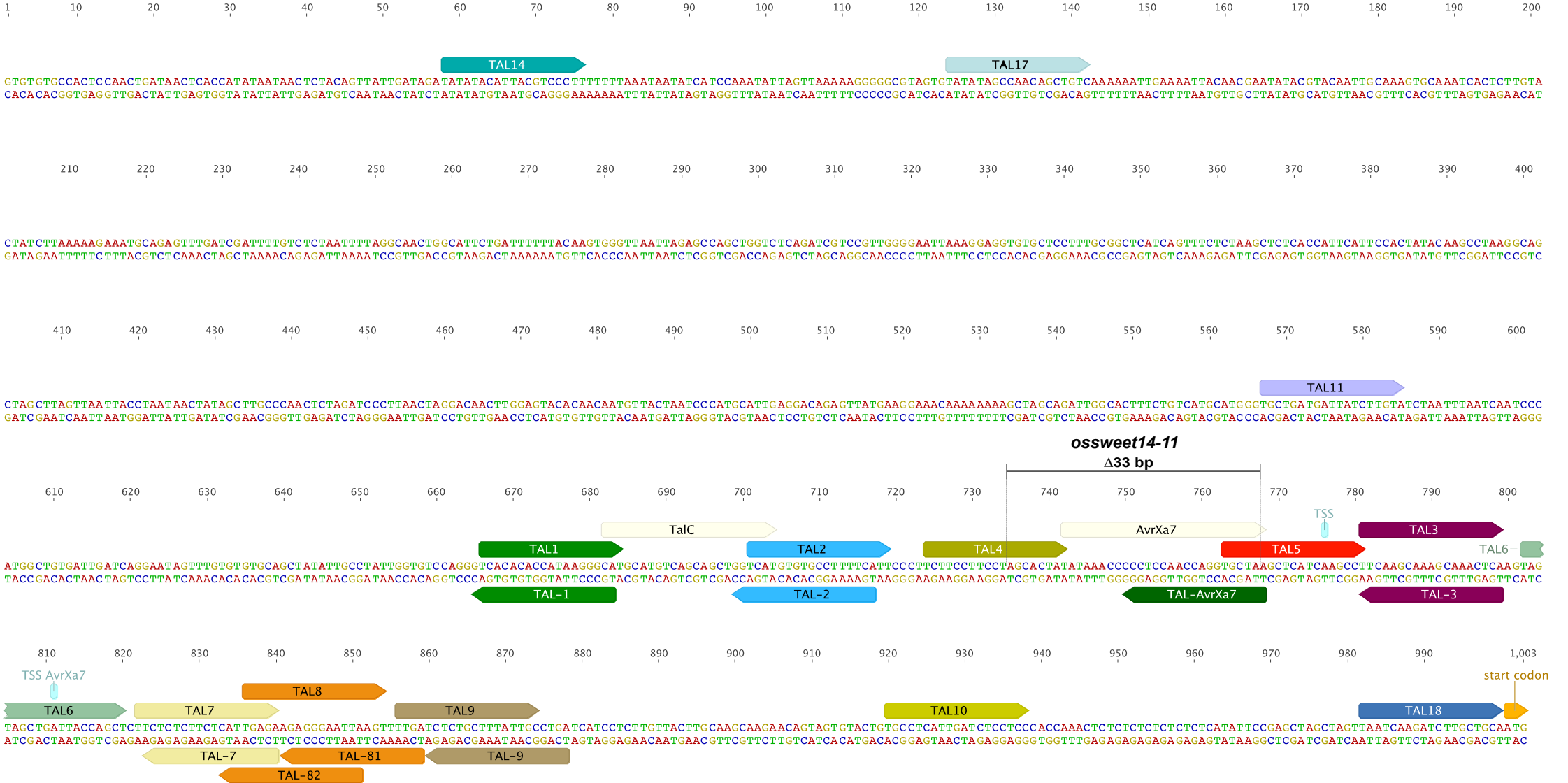

Supplement: S2 Fig — Overview of the OsSWEET14 promoter fragment 1kb upstream of the ATG. The binding sites and binding orientation of artificial TALEs (coloured) and the natural TALEs TalC and AvrXa7 (white) is marked with arrows. Reverse binding TALEs are labeled with "-" in front of the number. The 33 bp deletion in the sweet14-11 promoter is indicated. (PDF) [file pone.0173580.s002.pdf]

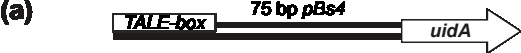

(b)

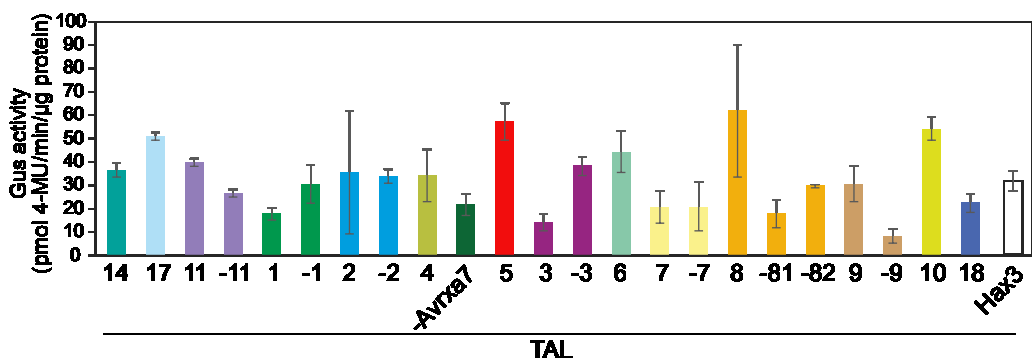

(c)

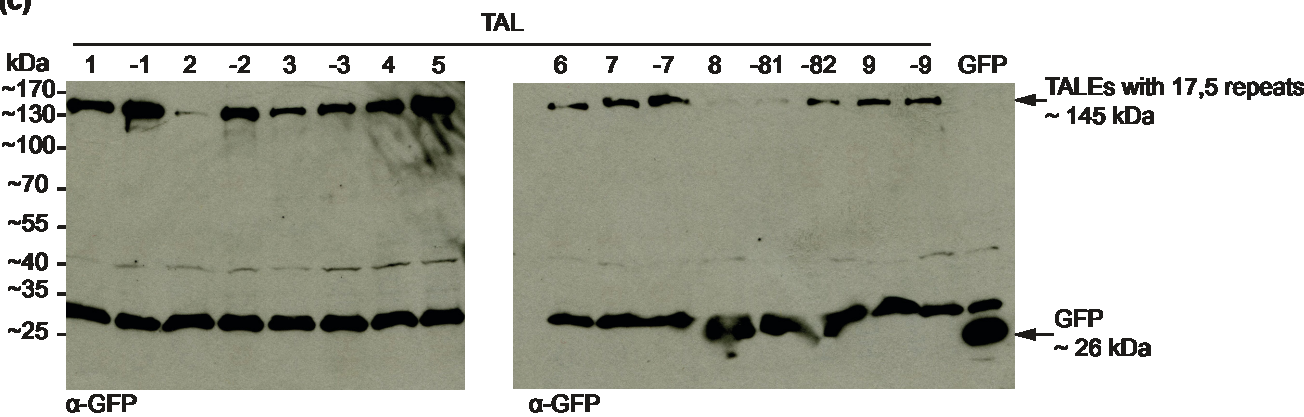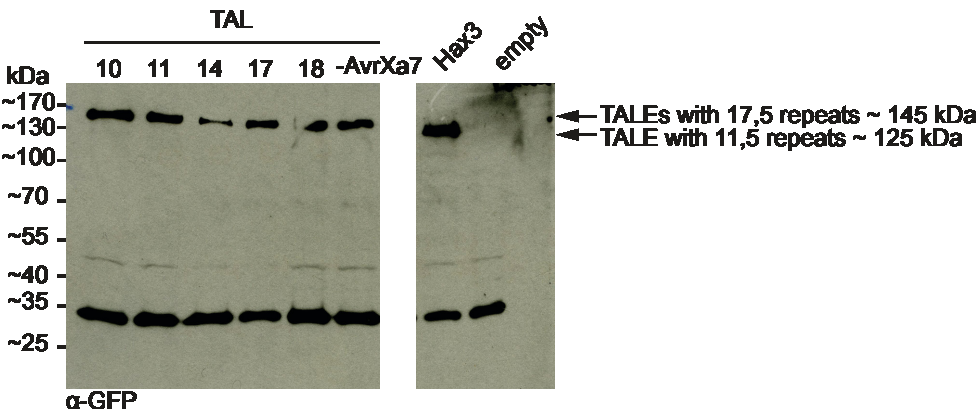

Supplement: S3 Fig — (A) Schematic overview of the reporter constructs. To compare the activation potential of each TALE independent of their position or orientation in the OsSWEET14 promoter their 18 bp TALE-boxes were inserted in forward orientation in front of the 75 bp pBs4 minimal promoter and a promoterless uidA reporter gene. (B) Activity of artificial and natural TALEs. Agrobacterium strains delivering the 35S-controlled TALE constructs and reporter constructs, respectively, were co-inoculated into N. benthamiana leaves. The TALE Hax3 and its Hax3-box reporter were used as positive control. The quantitative β-glucuronidase measurement was performed 2 dpi. Error bars were calculated on the basis of three independent replicates (4-MU, 4-methyl-umbelliferone). (C) Detection of TALE proteins in N. benthamiana. In parallel to the β-glucuronidase assay, six leaf discs of the inoculated area were harvested to analyze protein levels of the TALEs in N. benthamiana. SDS-PAGE followed by immunoblotting with an anti-GFP antibody directed against the N-terminal GFP that was fused to all analyzed TALEs shows a stable protein production. The expected size of artificial TALEs with 17.5 repeats and Hax3 with 11.5 repeats is indicated in kDa. The expression of GFP served as positive control, the sample with not-inoculated plant material (empty) as negative control. (PDF) [file pone.0173580.s003.pdf]

(a)

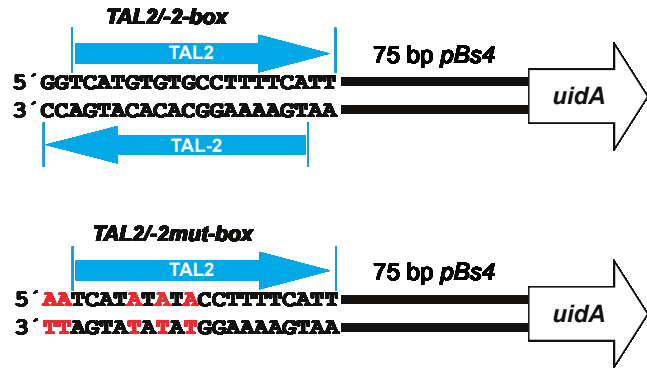

(b)

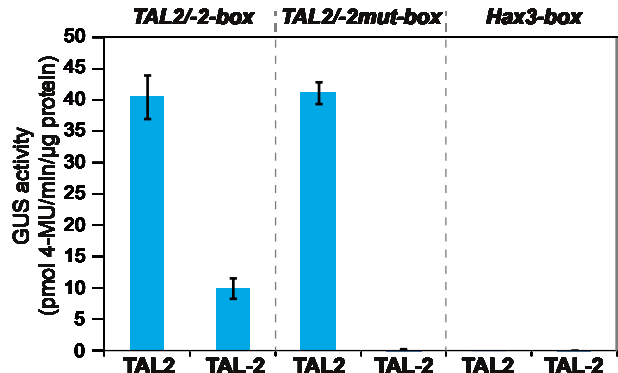

Supplement: S4 Fig — (A) Overlapping target sequences of the TALEs TAL2 and TAL-2 within reporter constructs. The TAL2/-2-box encompassing both TALE-boxes and the TAL2/-2mut-box with point mutations (red letters) that interfere only with binding of TAL-2, but not TAL2 were fused to the minimal promoter (75 bp Bs4) and a promoterless uidA reporter gene. (B) The activities of the TALEs were determined by quantitative β-Glucuronidase (GUS) measurement in N. benthamiana. Agrobacterium strains delivering the 35S controlled TALE constructs and the corresponding reporter constructs, respectively, were co-inoculated into N. benthamiana leaves. The TALE Hax3 and its Hax3-box reporter construct were used as control. GUS measurement were performed 2 dpi, error bars were calculated on the basis of three independent replicates (4-MU, 4-methyl-umbelliferone). (PDF) [file pone.0173580.s004.pdf]

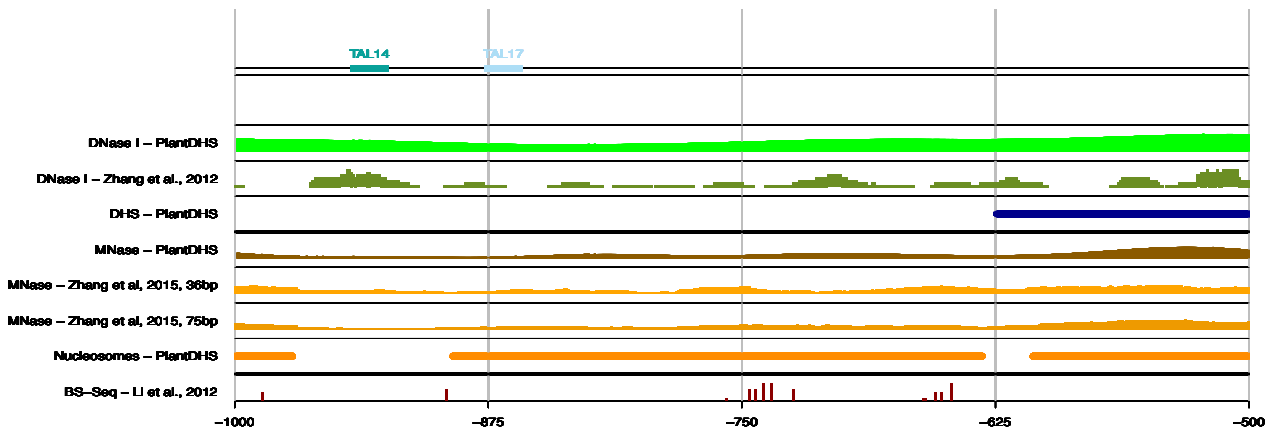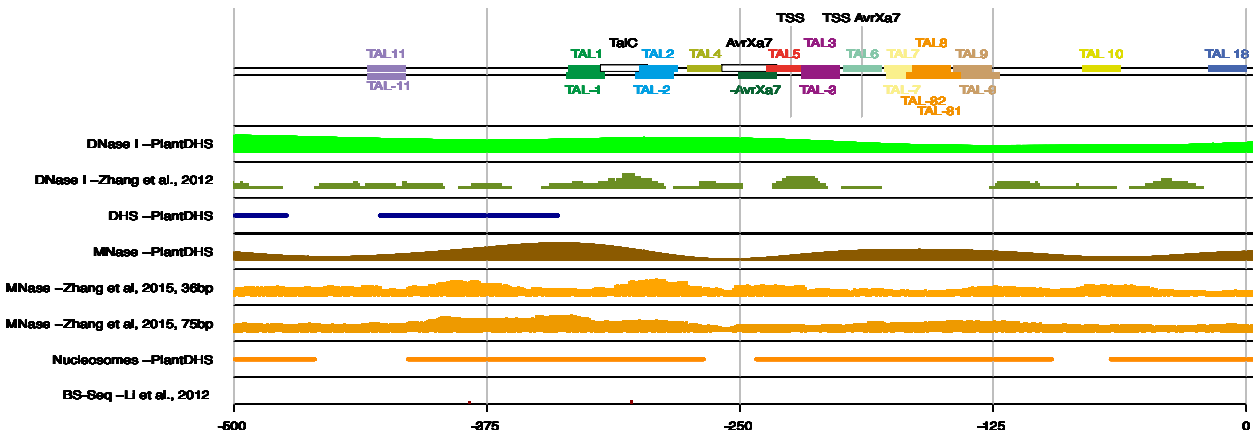

Supplement: S5 Fig — The 1 kb fragment of the OsSWEET14 promoter including the location of TALE-boxes (coloured boxes) was aligned to DNase I, MNase or bisulfite sequencing profiles indicating occupied promoter regions. DNase I and MNase profiles for rice leaves were downloaded from PlantDHS (http://plantdhs.org/Download, Zhang et al., 2015) in bigwig format. In addition, DNase I hypersensitive sites (DHS) and nucleosome tracks were downloaded from PlantDHS in gff format. DNase I reads of rice seedlings from (Zhang et al., 2012) where downloaded from NCBI Sequence Read Archive (SRA), accession SRX038423, and mapped to the rice MSU7/TIGR7 genome using bowtie2 (Langmead & Salzberg, 2012) with seed length 15 and at most 1 seed mismatch. MNase-Seq data from (Zhang et al., 2015b) of length 75 bp (SRR1536134) and 36 bp (SRR1536112) where also downloaded from SRA and mapped using bowtie2 with identical parameters. Bisulfite sequencing (BS-Seq) data of rice panicles from (Li et al., 2012) were downloaded from SRA (SRR037418, SRR037419, SRR037421, and SRR037422) and nucleotide-wise methylation percentages where determined using Bismarck (Krueger & Andrews, 2011) with bedGraph output. (PDF) [file pone.0173580.s005.pdf]
