# Supplementary material for: Dissection of TALE-dependent gene activation reveals that they induce transcription cooperatively and in both orientations
Source: PLoS One. 2017 Mar 16;12(3):e0173580. doi: 10.1371/journal.pone.0173580 (PMC5354296; doi:10.1371/journal.pone.0173580)
Supplement: S1 Table — (DOCX) [file pone.0173580.s006.docx]

# S1 Table. Oligonucleotides used in this study.

| **Oligo** | **Sequence (5'-3')** | **Application** |
| --- | --- | --- |
| Actin RT F | TGTGTGTGACAATGGAACTGGC | qRT |
| Actin RT R | GAGTCCAACACGATACCAGTTG | qRT |
| Os11N3 RT F | CTACCTGGCCCCACTGC | qRT |
| Os11N3 RT R | GTGCGCACCACCAGCC | qRT |
| 1kb pOs11N3 F | TTTGGTCTCACACCGTGTGTGCCACTCCAACTGATAAC | Amplification of the 1kb *OsSWEET14* promoter fragment |
| 1kb pOs11N3 R | TTTGGTCTCACCTTTGCAGCAAGATCTTGATTAACTAG | Amplification of the 1kb *OsSWEET14* promoter fragment |
| GUS 5´RACE T60 | GAACTGATCGTTAAAACTGCCTGGCAC | 5´RACE to determine transcriptional start sites in *N. benthamiana* |
| CTH3-GG-F | TTTGGTCTCAGGTGGAAGCATTGTTGCCCAGTTATC | Fusion of the C-terminal domain of Hax3 to dCas9 |
| CTH3-GG-R | TTTGGTCTCTAAGCTCACTGAGGCAATAGCTCCATC | Fusion of the C-terminal domain of Hax3 to dCas9 |
| Link F | TTTGGTCTCATTCGGGTGGCGGTAGTGGTGGCGGTGTGAGACCAAA | Fusion of the C-terminal domain of Hax3 to dCas9 |
| Link R | TTTGGTCTCACACCGCCACCACTACCGCCACCCGAATGAGACCAAA | Fusion of the C-terminal domain of Hax3 to dCas9 |
