# Supplementary material for: Dissection of TALE-dependent gene activation reveals that they induce transcription cooperatively and in both orientations
Source: PLoS One. 2017 Mar 16;12(3):e0173580. doi: 10.1371/journal.pone.0173580 (PMC5354296; doi:10.1371/journal.pone.0173580)
Supplement: S2 Table — (DOCX) [file pone.0173580.s007.docx]

# S2 Table. Artificial TALEs, their RVD sequences, and target DNA sequences.

^1^ Amino acid sequence in one letter code.

^2^ Forward DNA strand of TALE target sequences (TALE-boxes). Each RVD specifies one base and the N-terminal TALE domain specifies an additional thymine.

| ***pOsSWEET14* TALE** | **RVD sequence^1^ and TALE-box^2^ in the *OsSWEET14* promoter** |
| --- | --- |
| TAL1 | HD-NI-HD-NI-HD-NI-HD-HD-NI-NG-NI-NI-NN-NN-NN-HD-NI-NG  T C A C A C A C C A C A A G G G C A T |
| TAL-1 | NH-HD-HD-HD-NG-NG-NI-NG-NH-NH-NG-NH-NG-NH-NG-NH-NI-HD  T G C C C T T A T G G T G T G T G A C |
| TAL2 | HD-NI-NG-NN-NG-NN-NG-NN-HD-HD-NG-NG-NG-NG-HD-NI-NG-NG  T C A T G T G T G C C T T T T C A T T |
| TAL-2 | NN-NI-NI-NI-NI-NN-NN-HD-NI-HD-NI-HD-NI-NG-NN-NI-HD-HD  T G A A A A G G C A C A C A T G A C C |
| TAL3 | NG-HD-NI-NI-NN-HD-NI-NI-NI-NN-HD-NI-NI-NI-HD-NG-HD-NI  T T C A A G C A A A G C A A A C T C A |
| TAL-3 | NN-NI-NN-NG-NG-NG-NN-HD-NG-NG-NG-NN-HD-NG-NG-NN-NI-NI  T G A G T T T G C T T T G C T T G A A |
| TAL4 | HD-NG-NG-HD-HD-NG-NG-HD-HD-NG-NI-NN-HD-NI-HD-NG-NI-NG  T C T T C C T T C C T A G C A C T A T |
| TAL5 | NN-HD-NG-NI-NI-NN-HD-NG-HD-NI-NG-HD-NI-NI-NN-HD-HD-NG  T G C T A A G C T C A T C A A G C C T |
| TAL6 | NI-NN-NG-NI-NN-HD-NG-NN-NI-NG-NG-NI-HD-HD-NI-NN-HD-NG  T A G T A G C T G A T T A C C A G C T |
| TAL7 | NG-HD-NG-HD-NG-HD-NG-NG-HD-NG-HD-NI-NG-NG-NN-NI-NN-NI  T T C T C T C T T C T C A T T G A G A |
| TAL-7 | HD-NG-HD-NI-NI-NG-NN-NI-NN-NI-NI-NN-NI-NN-NI-NN-NI-NI  T C T C A A T G A G A A G A G A G A A |
| TAL8 | NN-NI-NN-NI-NI-NN-NI-NN-NN-NN-NI-NI-NG-NG-NI-NI-NN-NG  T G A G A A G A G G G A A G G A A G T |
| TAL-81 | HD-NI-NI-NI-NI-HD-NG-NG-NI-NI-NG-NG-HD-HD-HD-NG-HD-NG  T C A A A A C T T A A T T C C C T C T |
| TAL-82 | NI-NI-NG-NG-HD-HD-HD-NG-HD-NG-NG-HD-NG-HD-NI-NI-NG-NH  T A A T T C C C T C T T C T C A A T G |
| TAL9 | NG-NN-NI-NG-HD-NG-HD-NG-NN-HD-NG-NG-NG-NI-NG-NG-NN-HD  T T G A T C T C T G C T T T A T T G C |
| TAL-9 | HD-NI-NN-NN-HD-NI-NI-NG-NI-NI-NI-NN-HD-NI-NN-NI-NN-NI  T C A G G C A A T A A A G C A G A G A |
| TAL10 | NN-HD-HD-NG-HD-NI-NG-NG-NN-NI-NG-HD-NG-HD-HD-NG-HD-HD  T G C C T C A T T G A T C T C C T C C |
| TAL11 | NN-HD-NG-NN-NI-NG-NN-NI-NG-NG-NI-NG-HD-NG-NG-NN-NG-NI  T G C T G A T G A T T A T C T T G T A |
| TAL-11 | NI-HD-NI-NI-NH-NI-NG-NI-NI-NG-HD-NI-NG-HD-NI-NH-HD-NI  T A C A A G A T A A T C A T C A G C A |
| TAL14 | NI-NG-NI-NG-NI-HD-NI-NG-NG-NI-HD-NN-NG-HD-HD-HD-NG-NG  T A T A T A C A T T A C G T C C C T T |
| TAL17 | NI-NG-NI-NG-NI-NN-HD-HD-NI-NI-HD-NI-NN-HD-NG-NN-NG-HD  T A T A T A G C C A A C A G C T G T C |
| TAL18 | NI-NI-NG-HD-NI-NI-NH-NI-NG-HD-NG-NG-NH-HD-NG-NH-HD-NI  T A A T C A A G A T C T T G C T G C A |
| TAL-AvrXa7 | NG-NI-NN-HD-NI-HD-HD-NG-NN-NN-NG-NG-NN-NN-NI-NN-NN-NN  T T A G C A C C T G G T T G G A G G G |
