# Supplementary material for: Dissection of TALE-dependent gene activation reveals that they induce transcription cooperatively and in both orientations
Source: PLoS One. 2017 Mar 16;12(3):e0173580. doi: 10.1371/journal.pone.0173580 (PMC5354296; doi:10.1371/journal.pone.0173580)
Supplement: S3 Table — (DOCX) [file pone.0173580.s008.docx]

# S3 Table. sgRNA sequences and deskgen score.

| **sgRNA name** | **target sequence in *pOsSWEET14*^1^** | **activity score^2^** |
| --- | --- | --- |
| sg19 | TGGCACTTTCTGTCATGCAT**GGG** | 59 |
| sg20 | ATCTAATTTAATCAATCCCA**TGG** | 57 |
| sg21 | TGTGCAGCTATATTGCCTAT**TGG** | 44 |
| sg1 | TCCAGGGTCACACACCATAA**GGG** | 57 |
| sg7 | TCTCTCTTCTCATTGAGAAG**AGG** | 55 |
| sgTC | AGGGCATGCATGTCAGCAGC**TGG** | 45 |
| sg-TC | TGCTGACATGCATGCCCTTA**TGG** | 30 |
| sgAx7B | TATATAAACCCCCTCCAACC**AGG** | 59 |
| sg-3 | CTTGAGTTTGCTTTGCTTGA**AGG** | 27 |

^1^ 20 nucleotide sgRNA target sequence including the PAM motif in bold

^2^ activity score determined with deskgen, range 0 to 100 with 100 representing the highest target

efficiency
