# Supplementary material for: Dissection of TALE-dependent gene activation reveals that they induce transcription cooperatively and in both orientations
Source: PLoS One. 2017 Mar 16;12(3):e0173580. doi: 10.1371/journal.pone.0173580 (PMC5354296; doi:10.1371/journal.pone.0173580)
Supplement: S1 References — (DOCX) [file pone.0173580.s009.docx]

S1 References

Engler C, Youles M, Gruetzner R, Ehnert TM, Werner S, Jones JD, Patron NJ, Marillonnet S. A Golden Gate Modular Cloning Toolbox for Plants. ACS Synthetic Biology. 2014;3: 839-843.

Krueger F, Andrews SR. Bismark: a flexible aligner and methylation caller for Bisulfite-Seq applications. Bioinformatics. 2011;27: 1571-1572.

Langmead B, Salzberg SL. Fast gapped-read alignment with Bowtie 2. Nat Methods. 2012;9: 357-359.

Lescot M, Déhais P, Thijs G, Marchal K, Moreau Y, Van de Peer Y, et al. PlantCARE, a

database of plant cis-acting regulatory elements and a portal to tools for in silico analysis of

promoter sequences. Nucleic Acids Res. 2002;30: 325–7.

Li X, Zhu J, Hu F, Ge S, Ye M, Xiang H, Zhang G, Zheng X, Zhang H, Zhang S, et al. Single-base resolution maps of cultivated and wild rice methylomes and regulatory roles of DNA methylation in plant gene expression. BMC Genomics. 2012;13: 1–15.

Ordon J, Gantner J, Kemna J, Schwalgun L, Reschke M, Streubel J, Boch J, Stuttmann J. Generation of chromosomal deletions in dicotyledonous plants employing a user-friendly genome editing toolkit. Plant J. 2016; doi: 10.1111/tpj.13319.

Patron NJ, Orzaez D, Marillonnet S, Warzecha H, Matthewman C, Youles M, Raitskin O, Leveau A, Farre G, Rogers C, et al. Standards for plant synthetic biology: a common syntax for exchange of DNA parts. New Phytol.2015;208: 13-19.

Zhang T, Marand A, Jiang JM. PlantDHS: a database for DNase I hypersensitive sites in plants. Nucleic Acids Res. 2015;44: D1148-D1153.

Zhang WL, Wu YF, Schnable JC, Zeng ZX, Freeling M, Crawford GE, Jiang JM. High-resolution mapping of open chromatin in the rice genome. Genome Research. 2012;22: 151-162.
